# OS-BREEZE: Oil Spills Boundary Red Emission Zone Estimation Using Unmanned Surface Vehicles

**DOI:** 10.3390/s24020703

**Published:** 2024-01-22

**Authors:** Oren Elmakis, Semion Polinov, Tom Shaked, Gabi Gordon, Amir Degani

**Affiliations:** 1 Technion Autonomous Systems Program, Technion—Israel Institute of Technology, Haifa 3200003, Israel; oren.elmakis@campus.technion.ac.il (O.E.); shakedtom@campus.technion.ac.il (T.S.); 2 CAMERI—Coastal and Marine Engineering Research Institute Ltd., Haifa 3200003, Israel; semion.polinov@gmail.com (S.P.); ggordon@campus.technion.ac.il (G.G.)

**Keywords:** unmanned surface vehicles, marine pollution, oil spill mapping, catastrophic event, environmental monitoring, remote sensing

## Abstract

Maritime transport, responsible for delivering over eighty percent of the world’s goods, is the backbone of the global delivery industry. However, it also presents considerable environmental risks, particularly regarding aquatic contamination. Nearly ninety percent of marine oil spills near shores are attributed to human activities, highlighting the urgent need for continuous and effective surveillance. To address this pressing issue, this paper introduces a novel technique named OS-BREEZE. This method employs an Unmanned Surface Vehicle (USV) for assessing the extent of oil pollution on the sea surface. The OS-BREEZE algorithm directs the USV along the spill edge, facilitating rapid and accurate assessment of the contaminated area. The key contribution of this paper is the development of this novel approach for monitoring and managing marine pollution, which significantly reduces the path length required for mapping and estimating the size of the contaminated area. Furthermore, this paper presents a scale model experiment executed at the Coastal and Marine Engineering Research Institute (CAMERI). This experiment demonstrated the method’s enhanced speed and efficiency compared to traditional monitoring techniques. The experiment was methodically conducted across four distinct scenarios: the initial and advanced stages of an oil spill at the outer anchoring, as well as scenarios at the inner docking on both the stern and port sides.

## 1. Introduction

Oil spill incidents, particularly those involving crude oil, cause extensive and long-lasting harm to marine ecosystems, hinder economic progress, and have significant implications for public health [1]. These oil spill incidents can arise from various causes, including the leakage of an oil pipeline, discharges from ships, and other unforeseen disasters [2,3]. A prime example of such a disaster is the Deepwater Horizon, a catastrophic event that unfolded in the Gulf of Mexico in 2010. This incident led to the spillage of over 800 million liters of oil, causing extensive and enduring damage to the marine ecosystem, and the repercussions of this disaster could potentially persist for up to a century [4]. Furthermore, in a comprehensive analysis of oil satellite images from 2014 to 2019, Dong et al. [5] found that approximately ninety percent of oil slicks were located within 160 km of shorelines aligned with major shipping routes, which suggests a strong correlation between maritime traffic and the occurrence of oil slicks.

Rapid response is critical in the event of an oil spill, as there is a limited window of opportunity for effectively managing the spill [6]. Due to the rapid expansion of oil spills, time is of the essence for effective containment, which often involves using oil booms, and remediation by pumping out the oil.

Traditionally, the detection and monitoring of oil spills have required the involvement of human experts and specialized equipment. Expert personnel engage in extensive inspection missions, during which they visually examine the water surface and collect in-situ samples. In contrast to the urgent nature of oil spill response, this process is time-consuming. Monitoring large areas poses additional challenges, requiring significant resources, and is often affected by weather conditions.

The emergence of remote sensing techniques, which utilize satellite, aircraft, or drone sensing [7,8,9,10], enable oil spill detection efficiently without the need for in situ sampling. Effective oil spill surveillance is essential for managing oil spill events, and remote sensing technology can help to identify spills early before they cause significant harm. Oil spills can be detected and monitored in real-time using passive optical and active radar or lidar sensors [9]. However, remote sensing by satellite is limited by its resolution and frequency, which causes detection faults or delayed detection of oil spills [11]. These may lead to an oil spill spreading over a larger area and potentially causing more damage to the marine ecosystem.

Aerial surveillance for detecting oil spills requires highly skilled and trained operators capable of interpreting images to identify oil spills [12]. Unmanned Aerial Vehicles (UAVs) have emerged as a cost-effective and readily deployable solution, enabling continuous monitoring of the sea surface, and capturing high-resolution imagery [13]. The research of remote sensing with UAVs primarily focuses on the detection phase, introducing novel methods to detect oil pollution. Recent studies underscore a notable progression in the field of oil spill detection, attributing these advancements primarily to the evolution of deep learning methodologies [14,15].

Various studies have explored oil spill detection using different sensing instruments [16]. For instance, Koirala et al. conducted experiments to examine the potential of using an RGB camera mounted on a drone for the detection of various oil types, including diesel oil and hydraulic oil [17]. Thomas et al. demonstrated the efficacy of combining an infrared Convolutional Neural Network (CNN) [18]. Similarly, Zongchen et al. presented a method involving hyperspectral sensing for oil spill detection, utilizing a classifier that relies on spectral characteristics and CNN for enhanced accuracy [19]. However, the use of UAVs for oil spill monitoring presents limitations, such as restricted battery capacity, limited payload capability, and range. These factors currently limit UAVs’ ability to comprehensively monitor large areas. While the detection of an oil spill is an essential phase, planning the oil spill sampling path for surveying is equally crucial. This task involves designing paths that enable efficient data gathering and sampling from the affected area, ensuring comprehensive monitoring and accurate delineation of the polluted zone (red zone).

Unmanned Surface Vehicles (USVs) offer a practical solution for oil spill monitoring because their high payload and substantial battery capacity enable continuous operation [20]. However, the limited Field of View (FoV) of USV presents a challenge. Therefore, integrating their capabilities with effective path-planning strategies is essential to ensuring efficient monitoring and containment of oil spills.

Coverage Path Planning (CPP) represents a straightforward approach to handling the task of monitoring oil spills. In this method, the agent navigates through the relevant area, ensuring that the entire region is systematically covered. Shaocheng et al. [21] demonstrated a CPP approach by incorporating the principles of the traveling salesman problem and the utilization of a self-organizing map. Additionally, Bowen et al. [22] presented a method based on a deep Q network for efficient CPP, which aims to achieve complete coverage of the polluted area, a process that often involves prolonged and dense sampling.

In this context, our recent research introduced an approach known as the Boundary Red Emission Zone Estimation (BREEZE) [23]. This path-planning method, BREEZE, proposes estimating the boundary of the contaminated area, in events of gas leakage using UAV platforms. Rather than conducting exhaustive mapping of the entire area, the BREEZE approach strategically focuses on following the boundary of the red zone.

This current manuscript introduces the Oil Spill-BREEZE (OS-BREEZE) method, a novel approach for USVs that employs real-time computer vision techniques to estimate the red zone of oil pollution. It is designed to trace the boundary of this zone, offering a rapid and accurate assessment of the entire polluted area while addressing the challenge of the USV’s limited FoV. We demonstrate the efficacy of OS-BREEZE through a synthesized experiment, evaluating its effectiveness against traditional sampling approaches using four common metrics. The key contribution of this study is outlined as follows:

1. OS-BREEZE, an efficient pollution sampling path planning method for a USV platform.
2. Mapping red zone method to estimate the extent of pollution.
3. An experimental framework for examining oil spill events.

Furthermore, the study’s results indicate a substantial reduction in path length while maintaining high accuracy in pollution assessment.

## 2. Framework and Methodology

This section outlines the basis of the study, including a general understanding of the system, the methodology for visual sensing, the setup for experiments, and the performance measures. The design of the experiments showcases various oil spill scenarios to assess the efficacy of our algorithmic solution.

### 2.1. System Overview

**Figure 1:**
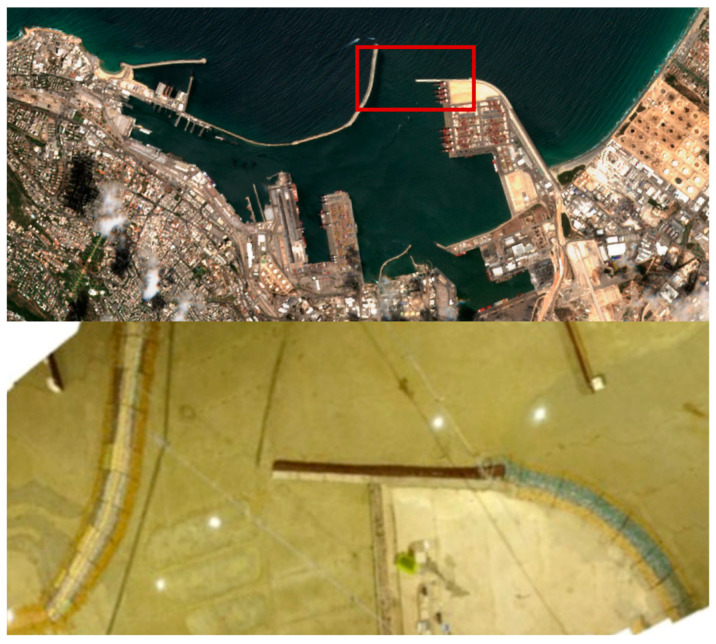
Aerial imagery for oil spill simulation. The top image depicts a satellite view of the Haifa Port, with a highlighted red rectangle indicating the focus area. Below is a stitched image of a scaled-down model (1:120 ratio) of the same section of the port, recreated in a controlled pool environment.

The system provides a comprehensive framework for evaluating the OS-BREEZE algorithm through small-scale experiments. These experiments encompass a complete experimental setup and software with an algorithmic workflow designed for boundary tracing, mapping, and determining termination criteria. The data were obtained from a drone (DJI phantom) platform, which conducted visual monitoring in the synthesized, scaled-down model of Haifa port, as illustrated in Figure 1. The software then processes the visual data collected by the drone, adapting them to closely simulate the visual capabilities of a USV platform.

### 2.2. Experimental Framework

The experimental setup depicted in Figure 1 features the Coastal and Marine Engineering Port Model (CPM), a detailed scaled model of Haifa port with a 1:120 ratio in three dimensions, including depth. The CPM was chosen for experimentation due to its accurate and detailed replication of the actual Haifa port, providing an ideal setting for simulating various oil spill scenarios. To mimic these scenarios, a dark substance was released from different sources and locations within the port and anchorage areas. 

To replicate a realistic scenario, the limited FoV of the USV was synthesized. This was achieved by cropping the entire imagery based on the simulated location of the USV and its visual inspection capabilities. This approach provided a more accurate representation of the USV’s visual abilities in real-world conditions.

### 2.3. Visual Sensor Model

**Figure 2:**
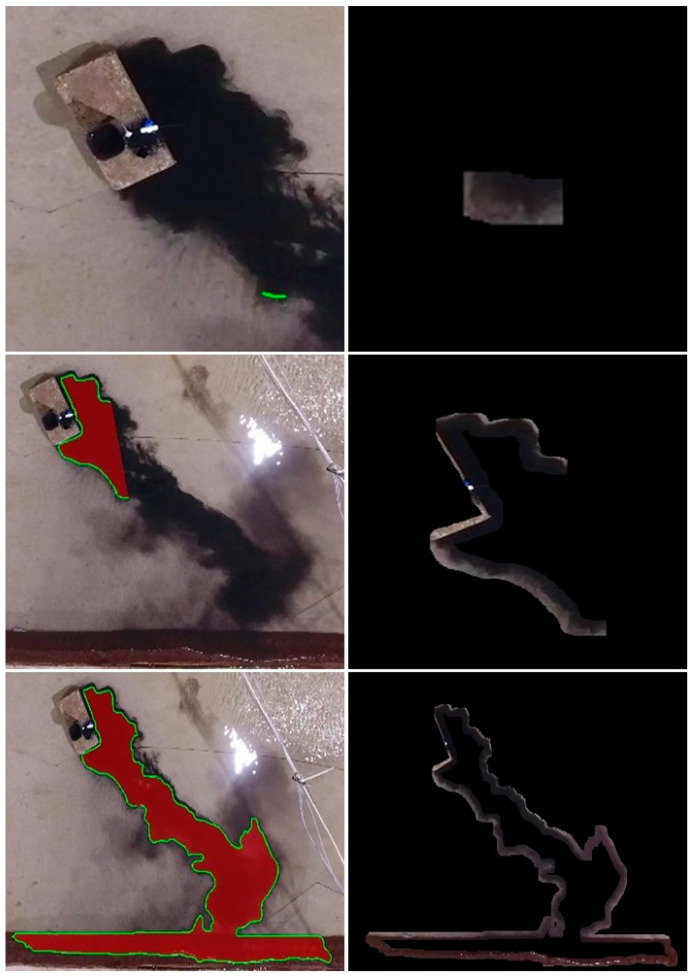
This composite image illustrates a sequence of steps depicting the process of a USV monitoring an oil spill based on the OS-BREEZE method.

The development of a visual model for a USV is essential for precisely estimating and enhancing the USV’s operational effectiveness. In this work, we treat the FoV of the USV as having a limited range, enabling it to inspect only a small area surrounding its current location. A represents the area that the USV can observe from its position, encompassing all the cells within its limited visual range. Figure 2 provides a sequence of steps depicting the process of the red zone estimation with OS-BREEZE. In the first image on the right, we observe the initial field of view of the USV, which captures only a portion of the affected area. As the monitoring process progresses, the subsequent images show the USV’s increasing estimation of the red zone.

### 2.4. Performance Measures

The study focuses on the task of estimating the red zone area, necessitating the accurate determination of the extent of the polluted area. Consequently, the performance of this task is evaluated by converting it into a classification task. In this setup, an agent is responsible for categorizing each area as either red cells or safe cells. This classification approach enables the utilization of established performance metrics to assess the effectiveness of the OS-BREEZE algorithm as demonstrated in [23]. The core components for measuring performance involve delineating the algorithm’s operational domain as a 2D cartesian grid, labeled M. This approach is theoretically grounded in spatial analysis theory, which advocates for a grid-based method to transform complex spatial environments into distinct, manageable units. Such a framework not only aids in the practical deployment of the OS-BREEZE algorithm but also offers an organized method to measure and interpret environmental data. Within the grid, the estimated red zone identified by the agent is represented as $M_{E}$, which is depicted in red in Figure 2. This illustrates the area estimated to be a red zone by the OS-BREEZE algorithm. The optimal red zone is denoted as $M_{O}$, representing the entire area identified as the ground truth red zone based on a predetermined threshold value.

This sets the stage for subsequent comparisons between the algorithm’s estimations and the optimal scenario, which are essential for evaluating the precision and accuracy of OS-BREEZE in identifying and delineating the extent of oil pollution. The grid is discretized, and the classification is categorized into three types: true-positive TP, where red zone cells are correctly identified; false-positive FP, where safe cells are incorrectly marked as red zone cells; and false negative FN, where polluted cells are incorrectly classified as safe.

Subsequently, the evaluation of the algorithm utilizes four performance measures: Precision, Recall, F1 score, and Coverage. Precision is defined as the ratio of true positive classification TP to the total number of positive classifications made by the algorithm, which is the sum of true positive and false positive TP+FP. This metric essentially quantifies the accuracy of positive predictions, indicating the proportion of correctly identified positive cases out of all cases labeled as positive by the algorithm.

(1) $$Precision=\frac{TP}{TP+FP}=\frac{M_{E}\cap M_{O}}{M_{E}}$$

Recall measures the proportion of true classifications TP to the total number of actual red zone cells, which is the sum of true positives and false negatives TP+FN. This metric reflects the algorithm’s ability to correctly identify all red zone cells in a given area. Essentially, it assesses the algorithm’s sensitivity in detecting the presence of red zone cells, indicating how well the algorithm avoids false negatives.

(2) $$Recall=\frac{TP}{TP+FN}=\frac{M_{E}\cap M_{O}}{M_{O}}$$

The F1 score is calculated as the harmonic mean of Precision and Recall, providing a balanced measure of the algorithm’s accuracy. Unlike a simple average, the harmonic mean tends to give more weight to lower values, which ensures that both Precision and Recall are reasonably high for a favorable F1 score.

(3) $$F1=\frac{TP}{TP+\frac{1}{2}\left(FN+TP\right)}=\frac{M_{E}\cap M_{O}}{M_{O}\cup M_{E}}$$

The final metric, Coverage, is computed by determining the ratio between the current estimated area, $M_{E}^{i}$, to the total estimated area, $M_{E}$, that is covered during the USV’s operational path. In this context, $M_{E}^{i}$, represents the extent of the area estimated as the red zone at a specific iteration step, i, during the USV’s task. The total estimated area, $M_{E}$, refers to the cumulative area classified by the algorithm over the entire course of action. This metric essentially assesses the algorithm’s capability to continuously and effectively classify areas, providing insight into its comprehensive operational coverage throughout the task.

(4) $$Coverage=\frac{M_{E}^{i}}{M_{E}}$$

## 3. Algorithmic Work

**Figure 3:**
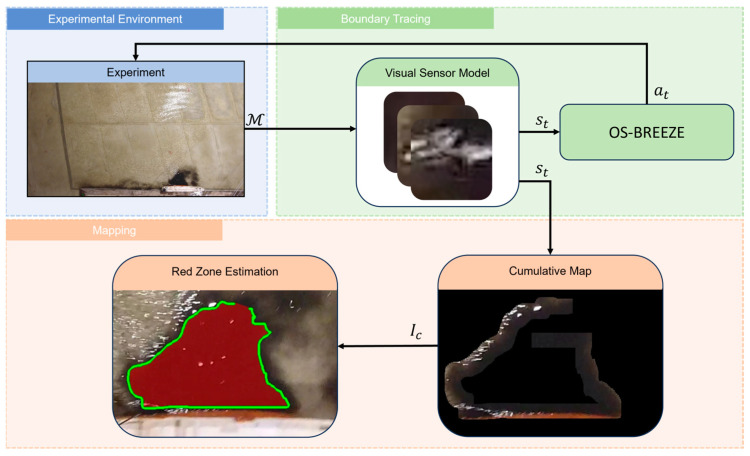
Schematic overview of the OS-BREEZE algorithm framework. This diagram is divided into three principal components: Experimental Environment, Boundary Tracing, and Mapping.

The following section offers an in-depth description of the OS-BREEZE algorithm, covering its various stages and operational framework. This is complemented by Figure 3, which graphically represents the algorithm’s structure and process flow. This flow involves three main components: Experimental Environment, Boundary Tracing, and Mapping.

Initially, the experimental environment provides the zone of contamination, denoted by M, informing the boundary tracing component’s visual sensor model. The visual sensor model then generates state visuals, $s_{t}$, for the OS-BREEZE algorithm, which in turn directs the agent’s actions via the command, $a_{t}$. During the mapping phase, these state visuals are compiled into a cumulative map. This map is subsequently employed in the red zone estimation process, which identifies and delineates the estimated contaminated area, labeled as $M_{E}$, thus effectively outlining the scope of the pollution.

### 3.1. OS-BREEZE

The OS-BREEZE, as outlined in Algorithm 1, is designed to effectively monitor dynamic oil spills, focusing particularly on tracing the evolving boundaries of these spills. At the core of its operation, the algorithm relies on inputs from the visual sensor model. This model is adept at simulating the FoV of the USV, thereby providing a realistic perspective of aquatic environment surveillance.

This visual, defined as $S_{t}$ and depicted in Figure 3 in the visual sensor model, is received by the OS-BREEZE as the input for the algorithm. The first step in the algorithm’s processing chain is the application of a Gaussian filter. This filter is applied through a convolution process, which involves overlaying the following Gaussian kernel onto the image:

(5) $$K\left(x,y\right)=\frac{1}{2\pi \sigma^{2}}e^{-\frac{x^{2}+y^{2}}{2\sigma^{2}}}$$

The convolution process is defined by the following function:

(6) $$S_{filtered}^{t}\left(u,v\right)=\sum_{i=-k}^{k}\sum_{j=-k}^{k}S_{t}\left(u+i,v+j\right)\cdot K\left(i,j\right)\;$$

In this context, $S_{filtered}^{t}\left(u,v\right)$ represents the output pixel value, k is the kernel size, $S_{t}$ is the state image, and $K\left(i,j\right)$ is the Gaussian kernel. This filter plays a role in mitigating noise within the visual data, which is a crucial step for ensuring accuracy. The noise in the image can stem from various sources, including environmental factors like tides and foam, as well as inherent sensor limitations.

Following noise reduction, the algorithm proceeds to implement a thresholding technique. In this step, a threshold filter is applied to the FOV visual. This filter is a binary-based filter with a predetermined opacity value that is characteristic of the water in the surveyed area. The purpose of this step is critical for distinguishing between areas affected by the oil spill and the surrounding water.

Subsequently, the algorithm advances to the stage where the Sobel filter is applied. This filter is crucial in the process of boundary detection, calculating the gradient magnitude and direction for each pixel in the image. The Sobel filter operates by applying two separate convolution kernels, horizontal $Sobel_{x}$ and vertical $Sobel_{y}$

(7) $$Sobel_{x}=\left[\begin{array}{ccc} -1 & 0 & 1\\ -2 & 0 & 2\\ -1 & 0 & 1 \end{array}\right],\;Sobel_{y}=\left[\begin{array}{ccc} -1 & -2 & -1\\ 0 & 0 & 0\\ 1 & 2 & 1 \end{array}\right]$$

These calculations of the Sobel gradient $I_{x},I_{y}$ enable the algorithm to effectively highlight the most significant changes in intensity, corresponding to the edges within the visual data.

In the algorithm’s final stage, the task is to trace the spill boundary, which involves calculating the perpendicular mean relative angle within the FoV image. This method accurately maps the contours of the spill. The calculation of the perpendicular mean relative angle represented by

(8) $$a_{t}=atan2\left(-\sum_{n}\frac{I_{x}\left(x,y\right)}{n},\;\sum_{n}\frac{I_{y}\left(x,y\right)}{n}\right)$$

This identifies the direction of edge gradients, facilitating the precise following of the spill boundary within the aquatic environment.
**Algorithm 1.** OS−BREEZE**Inputs:**$S_{t}\leftarrow USV\;FoV$**Output:**$a_{t}\leftarrow$ action$S_{filtered}^{t}\leftarrow K\left(x,y,\sigma \right)*S_{t}$for each (x,y) in $S_{filtered}^{t}$ do: if $S_{filtered}^{t}\left(x,y\right)\geq \epsilon$ do:  $I\left(x,y\right)=1$ else:  $I\left(x,y\right)=0$for each (x,y) in I: $I_{x}\left(x,y\right)\leftarrow {Sobel}_{x}*I$ $I_{y}\left(x,y\right)\leftarrow Sobel_{y}*I$$a_{t}\leftarrow atan2\left(-\sum_{n}\frac{I_{x}\left(x,y\right)}{n},\;\sum_{n}\frac{I_{y}\left(x,y\right)}{n}\right)$return $a_{t}$

### 3.2. Mapping and Red Zone Estimation

The mapping process, as shown in Algorithm 2, is initiated simultaneously with the boundary tracing, utilizing visuals received from the visual sensor. This process involves integrating the USV’s precise location data with the visuals of its surrounding area, as depicted in Figure 3. These visuals are then united into the cumulative map, $I_{c}$, encompassing all visuals collected from the locations visited by the USV.

Subsequently, the red zone estimation process takes over, utilizing the cumulative map to accurately estimate the spread of the red zone. This process mirrors the initial steps of the OS-BREEZE algorithm, starting with noise cancellation filtering to enhance estimation precision. Following this, a threshold filter is applied to classify the visuals based on the opacity values, a crucial step in identifying areas of the oil spill.

The next critical step involves locating boundary pixels within the exposed areas of the cumulative map. This is achieved by applying the Sobel filter and identifying pixels with a gradient magnitude above a certain threshold as potential boundary markers. Finally, the contour of the oil spill is delineated using the Topological Structural Analysis method [24], extracting it from the set of identified boundary pixels.
**Algorithm 2.** Red Zone Estimation**Inputs:**$I_{c}\leftarrow$ USV FoV**Output:**$M_{E}\leftarrow Estimated\;red\;zone$$I_{c,filtered}^{t}\left(x,y\right)\leftarrow G\left(x,y,\sigma \right)*I_{c}$for each (x,y) in $I_{c,filtered}^{t}$ do: if $I_{c,filtered}^{t}\left(x,y\right)\geq \epsilon$ do:  $I\left(x,y\right)=1$ else:  $I\left(x,y\right)=0$for each (x,y) in I: $I_{x}\left(x,y\right)\leftarrow {Sobel}_{x}*I$ $I_{y}\left(x,y\right)\leftarrow Sobel_{y}*I$ $M(x,y)\leftarrow \left\|\left(I_{x},I_{y}\right)\right\|$ if $M\left(x,y\right)\geq \;\epsilon_{mag}$ do: $B\leftarrow \left\|\left(I_{x},I_{y}\right)\right\|$$M_{E}\leftarrow contour\;Detection\left(B\right)$return $M_{E}$


## 4. Results

**Figure 4:**
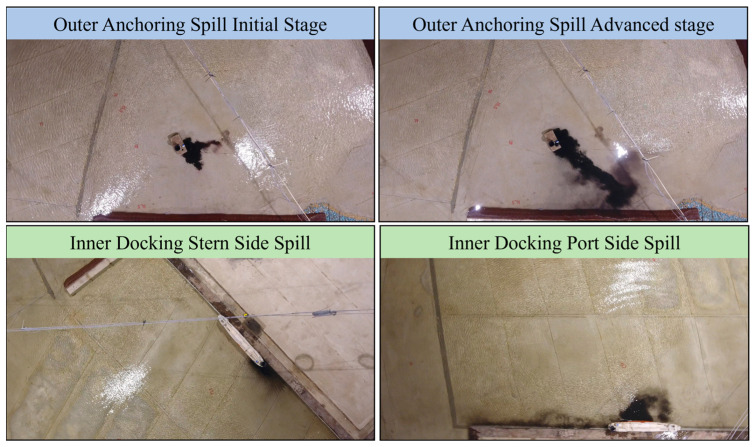
Demonstration of oil spill variations in a controlled experiment. The top images illustrate the spill’s development over time, showcasing the initial and advanced stages. The bottom images depict the challenges posed by spills along the vessel and the pier. Each image captures the unique dynamics and spread patterns of oil spills in various port spill scenarios, serving as test cases for the OS-BREEZE algorithm.

The results were obtained during a physical experiment, as presented in Figure 4 carried out on the CPM, an accurate scale model of Haifa port with an aspect ratio of 1:120. The CPM is designed to simulate real port scenarios, such as oil pollution.

In our experiment, we examined the OS-BREEZE algorithm framework’s performance in managing oil spills from various sources.

We experimented with different pollution scenarios, each varying in the source of pollution (vessel, pipeline), the rate of input (slow, medium, high), and changing meteorological and oceanographic conditions. Figure 4 presents four distinct spill scenarios: Outer Anchoring Spill Initial Stage, Outer Anchoring Spill Advanced Stage, Inner Docking Stern Side Spill, and Inner Docking Port Side Spill.

**Figure 5:**
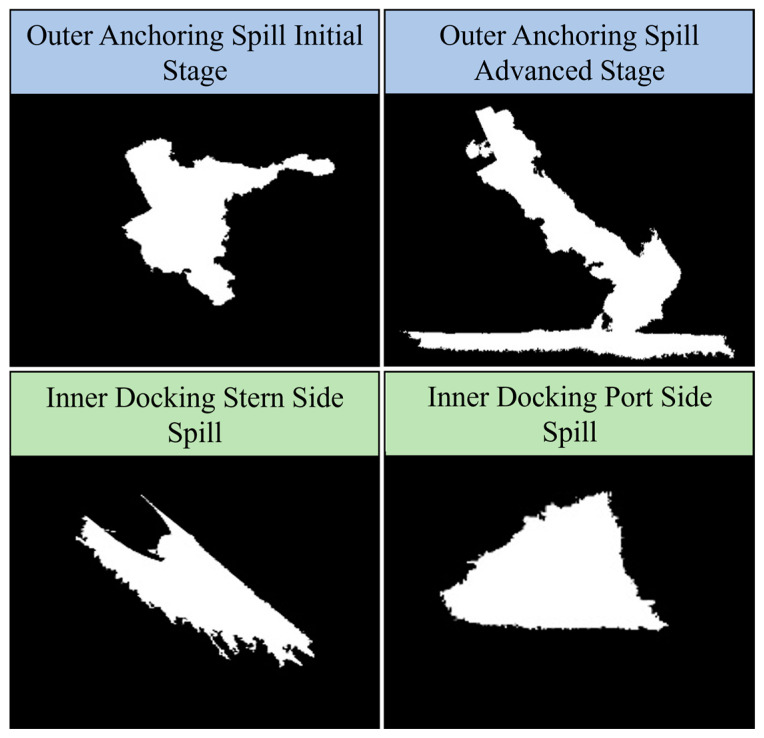
Presents the ground truth for red zone areas in experimented scenarios.

The Outer Anchoring Spill Initial Stage represents the early phase of a spill, where an oil pipeline lies on the seabed in the port area. In the Outer Anchoring Spill Advanced Stage, the USV encounters a more advanced phase of the spill, where the red zone of the oil spill has extended, making the estimation process more complex. Lastly, the Inner Docking spills illustrate the challenges posed by oil spills that spread along the vessel and the pier. Figure 5 presents the ground truth areas of the red zone for the examined scenarios, defined by a predetermined opacity level that serves as the threshold for pollution level in the experiment. The effectiveness of the OS-BREEZE algorithm in estimating the red zone was assessed by comparison with a standard baseline algorithm, known as Sweep, across a variety of scenarios. The Sweep algorithm, commonly likened to the path of a lawnmower traversing a lawn, is a systematic method frequently employed for comprehensive area coverage. Utilizing this method, the agent starts at one corner of the target area, and proceeds in a straight line until it encounters the boundary. Upon reaching this limit, the agent adjusts its position vertically by a predetermined distance and then continues in the opposite direction. This back-and-forth process is repeated in an exhaustive manner until the entire area is covered.

**Figure 6:**
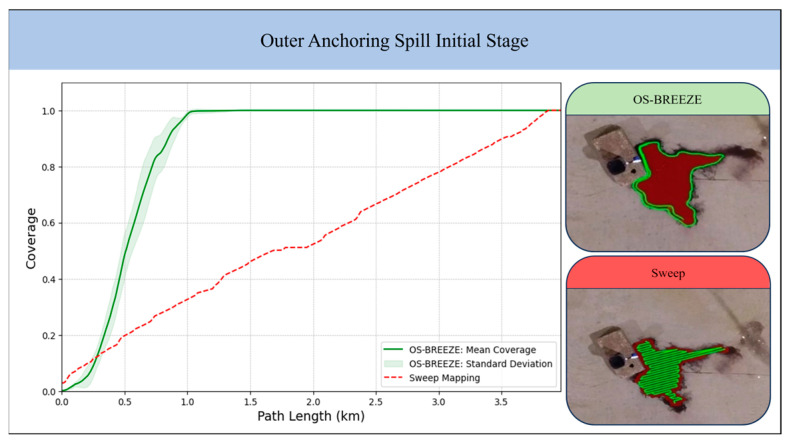

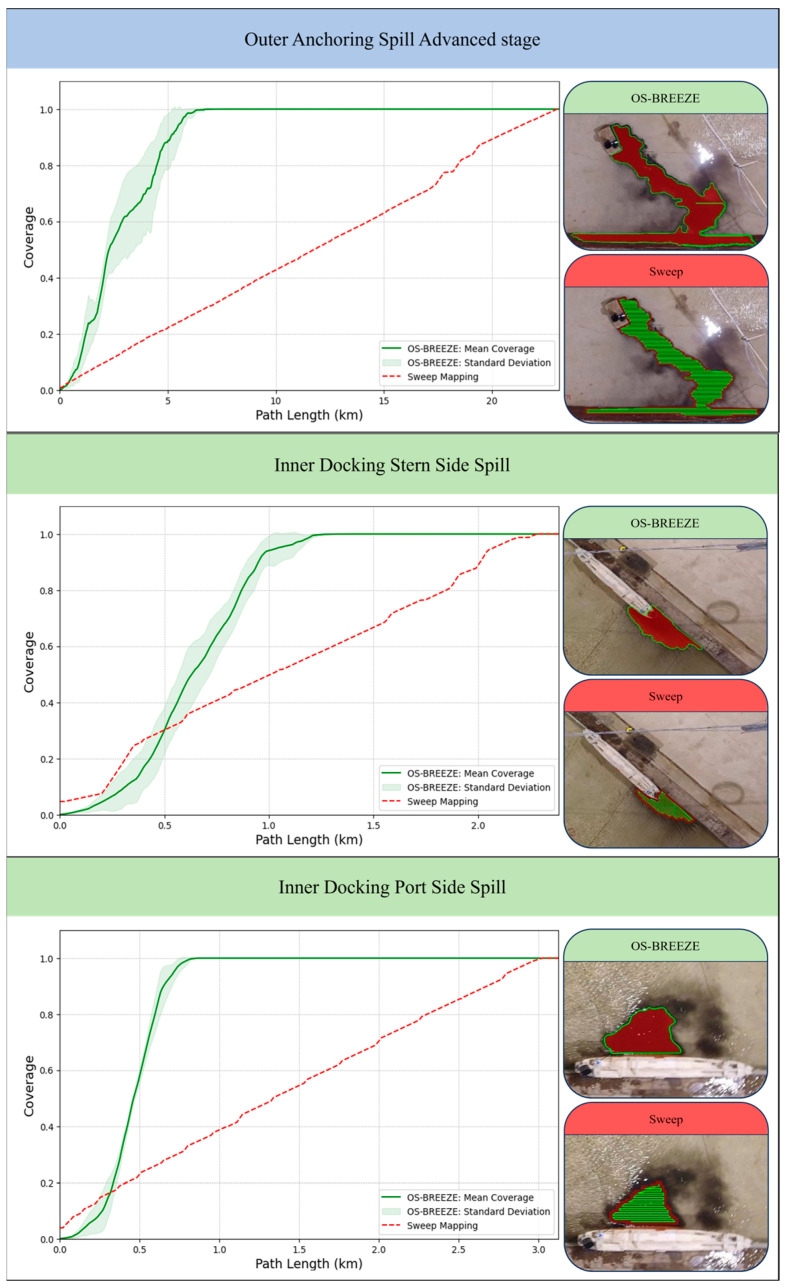
Comparative Analysis of OS-BREEZE and Sweep methods for oil spill monitoring. The figure presents a series of images and graphs for the Outer Anchoring Spill in its initial and advanced stages, followed by the Inner Docking Stern Side and Port Side spills. The graphs plot coverage against path length (km) indicating the efficiency of the methods and illustrating the path patterns of both methods.

This comparative analysis included evaluating the coverage of the path planning and comparing the path lengths generated by both the Sweep and OS-BREEZE algorithms, as illustrated in Figure 6. The results for the OS-BREEZE algorithm were derived from 10 separate runs, each under different initial conditions, where the mean was calculated as the following $\mu =\frac{\mu_{i}}{10}$ and the standard deviation (SD) calculated according to $\sigma =\sqrt{\frac{\sum {\left(\mu -\mu_{i}\right)}^{2}}{10}}$.

### Experimental Results

The experimental results for the Outer Anchoring and Inner Docking scenarios, using a USV deployed to map and monitor areas impacted by the oil spill, with the representation of the red zone ground truth oil spill is shown in Figure 5. The performance of the OS-BREEZE algorithm, as quantitatively measured by Precision, Recall, and F1-Score, is detailed in Table 1. This table provides a statistical analysis of the algorithm’s effectiveness across different scenarios, illustrating the mean (μ) and SD (σ).

The experimental results for the Outer Anchoring scenario are visualized in Figure 6, which compares the Sweep method with the OS-BREEZE. These results illustrate the differences in efficiency and coverage between the two methods through both graphical and visual data. OS-BREEZE is notable for its swift coverage rate and shorter path length in both the initial and advanced stages, suggesting a higher efficiency. In contrast, the Sweep method presents a slower coverage rate, indicating the need for a longer path to achieve similar coverage. The initial stage results specifically highlight OS-BREEZE’s ability to fully cover the area with a 1 km path, while Sweep requires about 3.75 km, and this efficiency remains apparent despite the SD. In the advanced stage, OS-BREEZE maintains its lead with a path length of about 5.6 km, in contrast to the Sweep’s substantially long 20 km path.

Visually, the two images compare the implementation of each method. OS-BREEZE is depicted as following a direct and focused path in green, closely followed by the spill’s boundary. In contrast, the Sweep method is characterized by a systematic, alternating coverage pattern. A notable observation is that in the initial stage, the Sweep covers a small section to the right of the red zone that OS-BREEZE does not.

The Inner Docking scenarios, shown in Figure 6, exhibit similar comparative results between the methods. In the Stern Side scenario, OS-BREEZE completes its coverage with a path length of about 1.5 km, whereas the Sweep requires about 2.5 km. In the Port Side scenario, OS-BREEZE’s path length is around 0.7 km, much shorter than Sweep’s path of 3 km. The graphs from both scenarios, underscored by Table 1, reveal OS-BREEZE’s boundary-following path and Sweep’s incremental coverage approach.

## 5. Discussion and Conclusions

The research presented herein introduces the OS-BREEZE algorithm, a path-planning strategy adept at delineating the red zone of oil pollution both swiftly and accurately. The efficiency and accuracy of OS-BREEZE are rigorously evaluated through comparative analyses against the conventional sweep method. These analyses were conducted across four varied pollution scenarios within a port environment, as detailed in Figure 6 and Table 1. Evaluation metrics for the OS-BREEZE method include Precision, Recall, and F1-Score. The findings consistently demonstrate that OS-BREEZE necessitates a reduced path length for effective red zone monitoring, thereby indicating a substantial improvement in operational efficiency compared to the traditional sweep method. Notably, the algorithm achieves an accuracy rate of approximately ninety percent, as measured across key metrics such as Precision, Recall, and F1 Score, as shown in Table 1. These results significantly underscore the precision and reliability of the OS-BREEZE algorithm, affirming its efficacy in conducting oil spill surveillance under diverse and challenging environmental conditions.

This study’s primary contribution, as highlighted by the findings, lies in the strategic approach of the OS-BREEZE algorithm, particularly its focus on delineating the boundary of the red zone of oil pollution. This approach contrasts with the conventional method that typically involves extensive coverage of the entire affected area. The novel strategy employed by OS-BREEZE allows for a significant reduction in the time required for monitoring. By strategically avoiding dense sampling of the entire polluted zone and instead concentrating on mapping the perimeter of the pollution, OS-BREEZE provides a more focused and resource-efficient method for surveillance.

Furthermore, this research employs a synthetic oil spill experiment, which requires further study in certain challenging conditions that are not accounted for in the experimental design. The potential influence of the USV on the distribution of the nearby oil spill. This influence encompasses factors such as the USV’s potential inaccuracies in sensing capabilities, which may compromise the precision of estimation. Additionally, various marine environmental phenomena, including waves and foam, can also impact the accuracy of the USV’s operations.

Moreover, the study utilizes a simulated camera model designed to replicate the visual capabilities of a USV in the scaled model. This model aids in visualizing the surrounding area. However, in real-world scenarios, several critical factors related to the camera must be considered for a more accurate representation. These include the camera’s resolution, its positioning, and the projection of the imagery.

## 6. Future Work

In forthcoming research efforts, the objective is to refine and extend the present methodology to facilitate a collaborative interface between UAV and USV, aiming to enhance overall efficacy. Whereas the current investigation is centered on monitoring applications, prospective studies will develop coordinated strategies for detecting and cleansing oil pollution in port areas. Incorporating oil spill detection and response functionalities into this collaborative framework is anticipated to significantly advance marine ecosystem protection and reduce the detrimental environmental impact of oil spillages.

Furthermore, this research will be expanded to investigate the boundary-following strategy under realistic marine conditions, including challenges such as foam and waves, which can only be authentically evaluated in an actual marine environment. A small-scale experiment will be developed to facilitate this investigation, utilizing a compact USV equipped with an onboard camera. This experimental setup is designed to examine various critical factors comprehensively. These include the impacts of camera positioning and tilting on the visual capabilities, potential inaccuracies in the USV’s sensing capabilities, and the influence of the USV’s presence on the distribution of the oil spill in its vicinity. By conducting these investigations, the study aims to gain a deeper understanding of how these elements interact and affect the efficiency and accuracy of the oil spill monitoring process.

## Figures and Tables

**Table 1:** Evaluation metrics for the OS-BREEZE method including Precision, Recall, and F1-Score.

Scenarios	Precision		Recall		F1-Score	
	μ	σ	μ	σ	μ	σ
Outer Anchoring Initial Stage	0.95	0.03	0.86	0.03	0.9	0.00
Outer Anchoring Advanced Stage	0.95	0.00	0.92	0.00	0.93	0.00
Inner Docking stern Side Oil Spill	0.9	0.06	0.88	0.07	0.89	0.02
Inner Docking Port Side Oil Spill	0.86	0.01	0.98	0.01	0.89	0.00

## Data Availability

Data are contained within the article.
